# Polysubstance Use Among Patients Treated With Buprenorphine From a National Urine Drug Test Database

**DOI:** 10.1001/jamanetworkopen.2021.23019

**Published:** 2021-09-10

**Authors:** Brendan Saloner, Penn Whitley, Leah LaRue, Eric Dawson, Angela Huskey

**Affiliations:** 1 Johns Hopkins Bloomberg School of Public Health, Baltimore, Maryland; 2 Millennium Health, San Diego, California

## Abstract

**Importance:**

Polysubstance use is a concern for patients treated for opioid use disorder (OUD). While buprenorphine can curtail harmful opioid use, co-occurring use of nonprescribed substances, such as cocaine, methamphetamine, and other opioids, may negatively affect treatment outcomes.

**Objective:**

To characterize factors associated with urine drug positivity for nonprescribed substances among patients prescribed buprenorphine.

**Design, Setting, and Participants:**

This cross-sectional study included patients who had been prescribed buprenorphine and who provided urine specimens for urine drug testing (UDT), as ordered by clinicians in primary care or behavioral health or at substance use disorder treatment centers, from 2013 to 2019. Specimens were analyzed by liquid chromatography–tandem mass spectrometry to assess positivity for several commonly used substances.

**Exposures:**

Buprenorphine prescription.

**Main Outcomes and Measures:**

Positivity for buprenorphine and several nonprescribed substances. Unadjusted trends in positivity for each nonprescribed substance were compared between specimens that did and did not test positive for buprenorphine. Multivariable logistic regression was used to examine factors associated with positivity; factors included patient age, sex, setting of care, payer, collection year, and census division.

**Results:**

The study included first UDT specimens from 150 000 patients, of whom 82 107 (54.74%) were men and 77 300 (51.53%) were aged 18 to 34 years. Across all specimens, 128 240 (85.49%) were positive for buprenorphine, and 71 373 (47.58%) were positive for 1 or more nonprescribed substances. From 2013 to 2019, positivity rates increased for most substances (eg, fentanyl: from 131 of 21 412 [0.61%] to 1464 of 13 597 [10.77%]). Factors associated with positivity varied widely by substance; for example, fentanyl positivity was highest for men (OR, 1.13; 95% CI, 1.06-1.21), patients aged 18 to 24 years (OR for patients ≥55 years, 0.46; 95% CI, 0.39-0.54), patients living in New England (OR, 1.19; 95% CI, 1.07-1.33), and patients with Medicaid (OR, 1.20; 95% CI, 1.11-1.31), whereas oxycodone positivity was greatest for women (OR for men, 0.84; 95% CI, 0.79-0.89), patients older than 55 years (OR, 1.42; 95% CI, 1.22-1.64), patients living in the South Atlantic (OR, 1.45, 95% CI, 1.33-1.58), and patients with private insurance (OR for Medicaid, 0.78; 95% CI, 0.73-0.84). Patients whose specimens were positive for buprenorphine were significantly less likely to be positive for other opioids (eg, fentanyl: OR for buprenorphine-negative samples, 6.71; 95% CI, 6.29-7.16; heroin: OR for buprenorphine-negative samples, 9.93; 95% CI, 9.31-10.59).

**Conclusions and Relevance:**

In this cross-sectional study, patterns of nonprescribed substance positivity among patients prescribed buprenorphine varied widely. This study highlights the utility of UDT in public health surveillance efforts related to patients treated with buprenorphine for OUD.

## Key Points

**Question:**

What are national trends in positivity for nonprescribed substances in urine drug tests (UDT) among patients prescribed buprenorphine?

**Findings:**

In this cross-sectional study of 150 000 urine specimens from patients prescribed buprenorphine, nearly half of patients tested positive for at least 1 nonprescribed substance. Compared with patients whose specimens were not positive for buprenorphine, those with positive specimens had significantly lower odds of testing positive for most nonprescribed substances, with the strongest associations for fentanyl and heroin.

**Meaning:**

These findings suggest that national UDT data can provide a unique window into changing polysubstance use trends among patients treated with buprenorphine.

## Teaser

This cross-sectional study characterizes factors associated with urine drug positivity for nonprescribed substances among patients prescribed buprenorphine.

## Introduction

Individuals with opioid use disorder (OUD) in the United States often use other substances. In national survey data from 2015 to 2016, 83% of all individuals with prescription OUD and 93% of people using heroin also self-reported either symptoms of alcohol use disorder or use of other substances.^1^ Marijuana was the most commonly reported substance used, but large proportions of patients also reported other drugs, particularly sedatives and cocaine. A national survey of people receiving substance use treatment from 2011 to 2018 found methamphetamine use increased most rapidly among people using opioids, while use of nonopioid prescription medications decreased.^2^ Self-reported methamphetamine use rose most rapidly among a national sample of patients initiating OUD treatment with medication between 1992 and 2017.^3^ Cocaine and methamphetamine–involved opioid overdose deaths have been rising.^4,5^ These trends are occurring alongside the emergence of potent illicit fentanyl, increasingly identified in combination with other substances.^6^

Changes in the drug use landscape pose a challenge for efforts to treat OUD. Patients with OUD who use other substances have lower retention in treatment and are more likely to relapse to substance use than those who only use opioids.^7,8,9^ Poorer outcomes among these patients likely reflect the greater complexity of need among these patients and the challenges of treating addiction to nonopioid substances. Whereas withdrawal and craving for opioids can be effectively treated with medications,^10^ there are no currently approved medications for addiction to substances such as cocaine or methamphetamine.

In an era of rising polysubstance use, it is important to identify illicit drug and alcohol use patterns among patients treated for OUD. The objective of our study was to characterize trends in nonprescribed substance use among people with OUD who initiate buprenorphine pharmacotherapy. We focus on buprenorphine because its use in OUD treatment has grown rapidly in recent years^11^ and is provided to a heterogeneous population of patients who receive care from both specialty clinics and office-based clinicians. Periodic urine drug testing (UDT) is frequently used by clinicians to monitor patient progress in treatment, to address potential medication safety issues from drug-drug interactions, and to adjust treatment plans (eg, requirements around frequency of follow-up visits).^12^ UDT can provide an important window into the changing substance use issues among patients treated with medications for OUD. UDT has been used to examine trends in substance use (not specific to patients treated for OUD).^6,13,14,15^ Furthermore, prior studies have examined UDT findings among OUD patients treated at single sites or hospital health systems.^9,16,17^ We are not aware of any studies that specifically examine national trends in definitive UDT findings among OUD patients. Definitive UDT findings can add important information to self-reported substance use from other studies because definitive UDT often provides greater specificity about types of drugs patients have been using, including drugs that they may not be aware of (eg, fentanyl adulterating cocaine or heroin).

## Methods

### Data Source and Sample Selection

We conducted a retrospective study of UDT results from January 1, 2013, to December 31, 2019, from patient specimens submitted for testing by health care practices (which includes specialty clinics and office-based clinicians). Included specimens were from patients aged 18 years or older who were identified by their clinician as being prescribed buprenorphine. We excluded a small number of cases (0.92%) with missing sex. A list of prescribed medications for each study participant was noted on the requisition form by the health care practice ordering the UDT. Specimens were collected from health care practices from all 50 states and the District of Columbia. Each UDT was individually ordered by the clinician based on medical necessity. A single specimen for each patient was selected based on the earliest specimen collection date, and repeated measurements were removed from the sample. The study used a convenience sample of 150 000 randomly selected patient specimens from Millennium Health’s proprietary UDT database with test orders for definitive UDT for buprenorphine. Specimens were analyzed by liquid chromatography–tandem mass spectrometry (LC-MS/MS) for each analyte. The LC-MS/MS testing method is a laboratory-developed test with performance characteristics determined by Millennium Health, which is certified by the Clinical Laboratory Improvement Amendments and accredited by the College of American Pathologists for high-complexity testing. The study protocol was determined to be exempt by the Johns Hopkins School of Public Health institutional review board, was approved by the Aspire independent review board, and included a waiver of consent for the use of deidentified data. This study followed the Strengthening the Reporting of Observational Studies in Epidemiology (STROBE) reporting guideline.

We examined analyte/metabolite results consistent with either illicit or nonprescribed substance use. All specimens were tested for buprenorphine (and its metabolite, norbuprenorphine). The following drugs and/or drug classes were tested for in a subset of patient specimens based on a clinician’s determination of medical necessity^12^ (with analytes and metabolites tested in parentheses): cocaine (benzoylecgonine), methamphetamine, fentanyl (fentanyl, norfentanyl), heroin (6-monoacetylmorphine), alcohol (ethyl glucuronide, ethyl sulfate), marijuana (delta-9-tetrahydrocannabinol carboxylic acid), methadone (methadone, 2-ethylidene-1,5-dimethyl-3,3-diphenylpyrrolidine), gabapentin, tramadol (tramadol, N-desmethyl-tramadol, O-desmethyl-tramadol), hydrocodone (hydrocodone, norhydrocodone), oxycodone (oxycodone, noroxycodone), and benzodiazepines (alpha-hydroxyalprazolam, 7-amino-clonazepam, nordiazepam, oxazepam, temazepam, lorazepam). If any parent analyte or metabolite within a drug class was detected, the drug or drug class was considered positive for that specimen. However, we excluded positive results for medications that were reported by clinicians to be currently prescribed to patients: 6348 of the 150 000 samples (4.23%) had reported medications for at least 1 of the 12 analytes and were removed from the analysis. Because not all specimens were tested for each analyte, we calculated rates out of all specimens tested for the analyte. (The number of positive tests and positivity rates for each analyte are shown in Table 1.)

**Table 1.** Characteristics of Urine Drug Testing Specimens Tested for Prescribed Buprenorphine Between January 1, 2013, and December 31, 2019

Characteristic	Specimens tested, No. (%)	
	Total	2015-2019 Specimens only^a^
Unique patient specimens	150 000 (100.00)	88 685 (100.00)
Sex		
Female	67 893 (45.26)	41 212 (46.47)
Male	82 107 (54.74)	47 473 (53.53)
Age, y		
18-24	15 151 (10.10)	7239 (8.16)
25-34	62 149 (41.43)	36 334 (40.97)
35-44	40 681 (27.12)	25 660 (28.93)
45-54	20 186 (13.46)	11 963 (13.49)
≥55	11 593 (7.73)	7387 (8.33)
US Census Division^b^		
East North Central	40 033 (26.69)	25 322 (28.55)
East South Central	12 483 (8.32)	8619 (9.72)
Mid-Atlantic	25 494 (17.00)	10 765 (12.14)
Mountain	9527 (6.35)	6324 (7.13)
New England	9666 (6.44)	6799 (7.67)
Pacific	13 384 (8.92)	10 684 (12.05)
South Atlantic	30 398 (20.27)	15 415 (17.38)
West North Central	3115 (2.08)	2462 (2.78)
West South Central	5900 (3.93)	2295 (2.59)
Health care practice specialty		
Behavioral health	22 575 (15.05)	16 123 (18.18)
Primary care physician	45 129 (30.09)	25 437 (28.68)
Substance use treatment	82 296 (54.86)	47 125 (53.14)
Payer group		
Medicaid	44 220 (29.48)	44 220 (49.86)
Medicare	7279 (4.85)	7279 (8.21)
Private insurance	23 725 (15.82)	23 725 (26.75)
Uninsured	12 395 (8.26)	12 395 (13.98)
Unknown^c^	62 381 (41.59)	1066 (1.20)
UDT positives^d^		
Buprenorphine	128 240 (85.49)	74 744 (84.28)
Benzodiazepines	22 484 (18.87)	12 350 (17.78)
Cocaine	12 037 (8.87)	8160 (10.22)
Alcohol	12 215 (14.67)	7473 (14.59)
Fentanyl	5217 (4.94)	4844 (6.96)
Gabapentin^e^	3773 (14.66)	3630 (15.05)
Heroin	6785 (5.38)	4774 (6.19)
Hydrocodone	5699 (4.19)	2942 (3.72)
Oxycodone	8586 (6.30)	4815 (6.03)
Methadone	2667 (2.07)	1442 (1.91)
Methamphetamine	8740 (6.85)	7683 (10.63)
Marijuana	24 564 (26.92)	19 138 (28.16)
Tramadol	1953 (2.53)	1521 (2.47)

Abbreviation: UDT, Urine Drug Testing.

^a^ Regression analysis was performed on the subset of samples collected from 2015 to 2019 because of missing payer group covariate information in 2013 and 2014.

^b^ US Census Divisions were as follows: East North Central (Illinois, Indiana, Michigan, Ohio, and Wisconsin); East South Central (Alabama, Kentucky, Mississippi, and Tennessee); Mid-Atlantic (New Jersey, New York, and Pennsylvania); Mountain (Arizona, Colorado, Idaho, Montana, Nevada, New Mexico, Utah, and Wyoming); New England (Connecticut, Maine, Massachusetts, New Hampshire, Rhode Island, and Vermont); Pacific (Alaska, California, Hawaii, Oregon, and Washington); South Atlantic (Delaware, Florida, Georgia, Maryland, North Carolina, South Carolina, Virginia, District of Columbia, and West Virginia); West North Central (Iowa, Kansas, Minnesota, Missouri, Nebraska, North Dakota, and South Dakota); and West South Central (Arkansas, Louisiana, Oklahoma, and Texas).

^c^ Overall, 61 315 of the 62 381 unknown specimens (98.3%) were found in the 2013 to 2014 time period.

^d^ All positive numbers and positivity rates are based on the nonprescribed analyte population except buprenorphine.

^e^ No gabapentin UDT results occurred before June 2014.

### Covariates

Additional characteristics for each specimen included the patient’s sex and age (discretized into 5 groups: 18-24 years, 25-34 years, 35-44 years, 45-54 years, and ≥55 years) and location of the health care clinician (9 major US Census divisions). We also included health care specialty and payer group. Specialty is a 3-level categorization designed to describe the clinical practice of the ordering clinician; it was initially chosen by Millennium Health and subsequently verified by the ordering clinician. Payer group is a 5-level classification of insurance and/or payer type, available beginning in 2015.

### Statistical Analysis

Annual trends in crude positivity rates were calculated as the percentage of tests that were positive in the sample in each year from 2013 to 2019. Clopper-Pearson 95% binomial CIs were estimated for the raw positivity rates. These rates and CI values were calculated per year and stratified by buprenorphine detection.

Logistic regression was performed to evaluate the association of demographic features with buprenorphine detection. Collection year, clinic location (US Census division), sex, age, payer group, and health care specialty were modeled as explanatory variables. All regression modeling was performed on data from 2015 to 2019 only because of missing payer group data before 2015. Type III analysis of deviance was performed to evaluate the significance of each model factor using likelihood ratio tests. Adjusted odds ratios (aORs) and 95% CIs for all factors in the logistic model were estimated. Marginal probabilities (least square means), Sidak-corrected 95% CI values, and Tukey-corrected *P* values for all pairwise comparisons were also estimated.

Similar regression models were also constructed for the nonbuprenorphine drugs and drug classes tested. In addition to collection year (2015-2019 only), US Census division, sex, age, payer group, and health care specialty, the detection status of buprenorphine (positive or negative) was added as an explanatory variable to each of the 12 independent regression models. Detection of the given drug class was treated as the dependent variable. Finally, since first specimens likely include patients who have not yet taken their first prescribed buprenorphine dose, we conducted a sensitivity analysis to examine positivity rates with other substances using second specimens (available for 137 722 specimens [91.8%]).

R version 4.0.2 (R Project for Statistical Computing) was used for data analysis. Statistical significance was set at *P* < .05; all tests were 2-tailed.

## Results

Table 1 displays the characteristics of the study sample of 150 000 specimens. Across years, 82 107 specimens (54.74%) were from male patients, and 77 300 (51.53%) were from patients younger than 35 years. Referring health care practices were most likely in the Census divisions of East North Central (40 033 [26.69%]) and South Atlantic (30 398 [20.27%]). More than half of referring health care practices were substance use disorder treatment centers (82 296 [54.86%]), followed by primary care (45 129 [30.09%]) and behavioral health (22 575 [15.05%]) practices. Most common payers for tested specimens were Medicaid (44 220 [29.48%]), private insurance (23 725 [15.82%]), Medicare (7279 [4.85%]), and uninsured (12 395 [8.26%]). The payer group was unknown for 62 381 specimens (41.59%). While most characteristics were similar across years (eTable 1 in the Supplement), most specimens with an unknown payer were collected in 2013 and 2014 (only 1066 [1.20%] of 2015-2019 specimens were from the unknown payor group category). The analytes we examined were tested for between 77 299 specimens (51.53%) for tramadol and 136 187 specimens (90.79%) for oxycodone. Of all specimens tested, 128 240 (85.49%) were positive for buprenorphine, and 71 373 (47.58%) were positive for at least 1 nonprescribed substance.

**Figure 1.**
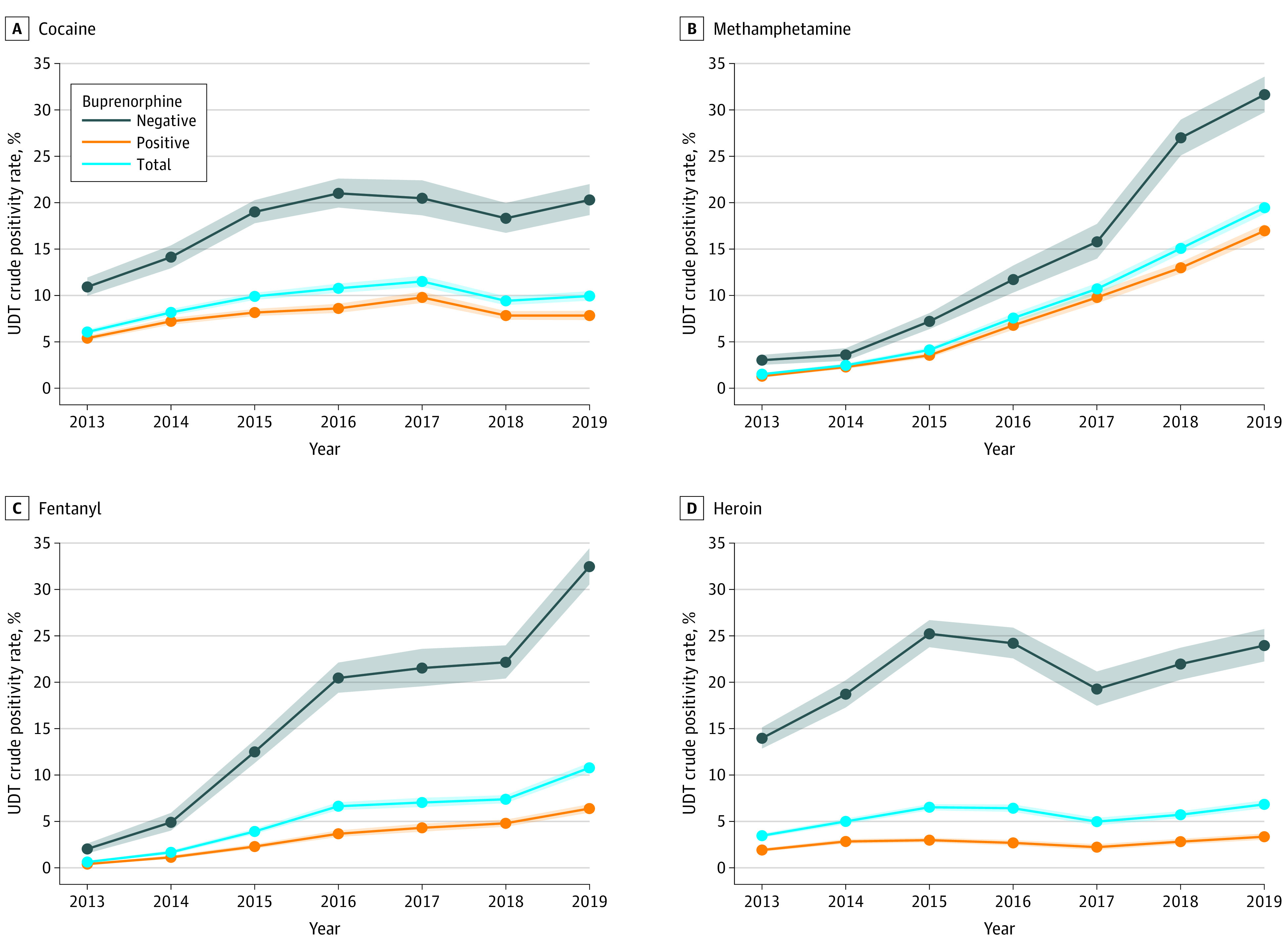
Positivity Rates for Cocaine, Methamphetamine, Fentanyl, and Heroin During the Study Period Shaded areas indicate 95% CIs.

Figure 1 shows trends in the overall average positivity rate for 4 example substances (cocaine, methamphetamine, fentanyl, and heroin) among UDT specimens from 2013 to 2019. Overall, positivity increased 63.7% for cocaine (from 2005 of 32 996 [6.08%] to 1356 of 13 627 [9.95%]), 1171.2% for methamphetamine (from 504 of 33 022 [1.53%] to 2663 of 13 693 [19.45%]), 1665.6% for fentanyl (131 of 21 412 [0.61%] to 1464 of 13 597 [10.77%]), and 97.4% for heroin (986 of 28 404 [3.47%] to 952 of 13 903 [6.85%]). Specimens positive for buprenorphine were consistently less likely to be positive for each illicit substance, but the gap differed by substance and year. In 2019, buprenorphine-positive specimens were 158.6% less likely to be positive for cocaine than buprenorphine-negative specimens (7.85% [95% CI, 7.36%-8.36%] vs 20.30% [18.68%-22.01%]), 86.4% for methamphetamine (16.96% [95% CI, 16.28%-17.67%] vs 31.62% [95% CI, 29.74%-33.56%]), 408.8% for fentanyl (6.38% [95% CI, 5.93%-6.84%] vs 32.46% [95% CI, 30.54%-34.42%]), and 613.1% for heroin (3.36% [95% CI, 3.04%-3.70%] vs 23.96% [95% CI, 22.25%-25.74%]). Corresponding data are found in eTable 2 in the Supplement, and trend plots for a larger set of substances are shown in the eFigure in the Supplement.

**Figure 2.**
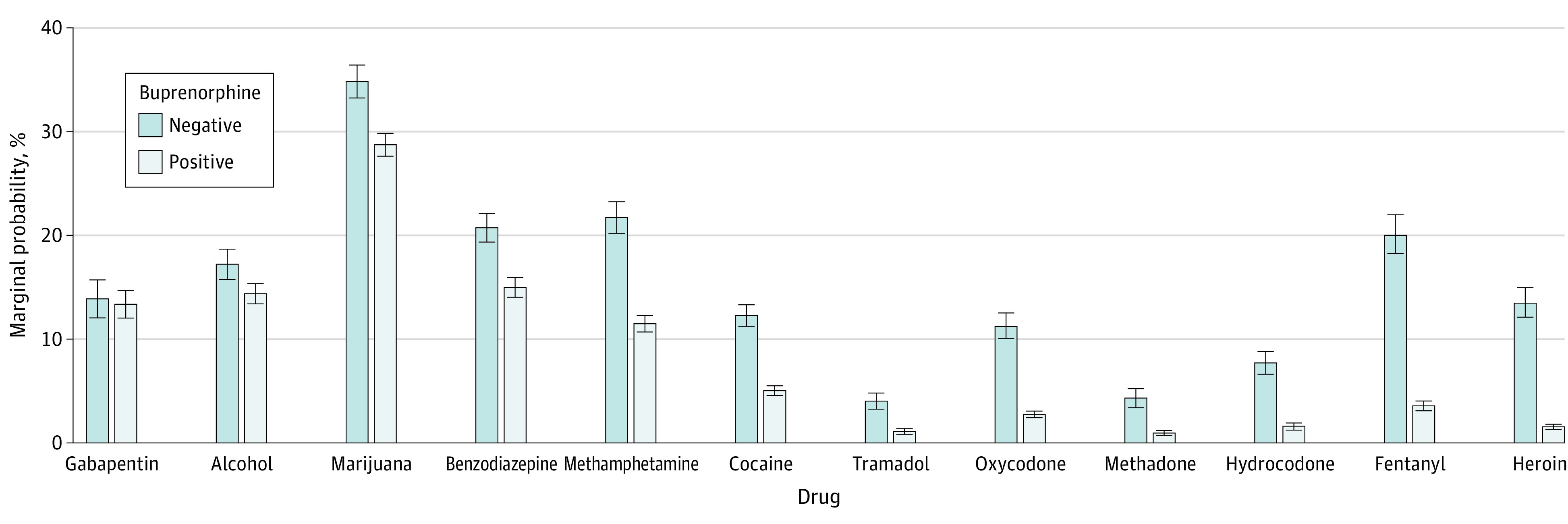
Probability of Testing Positive for Nonprescribed Substance by Buprenorphine Positivity. Error bars indicate 95% CIs.

Marginal probabilities and ORs for detection of other substances in buprenorphine-negative vs buprenorphine-positive specimens collected in 2019 are shown in Figure 2. Consistent with the unadjusted data, aORs were highest in buprenorphine-negative specimens for each of the substances detected. All aORs were significantly greater than 1 with the exception of gabapentin (aOR 1.05, 95% CI, 0.94-1.15). Other aORs were 1.24 (95% CI, 1.16-1.32) for alcohol, 1.33 (95% CI, 1.27-1.39) for marijuana, 1.48 (95% CI, 1.41-1.56) for benzodiazepines, 2.14 (95% CI, 2.01-2.27) for methamphetamine, 2.64 (95% CI, 2.50-2.78) for cocaine, 3.69 (95% CI, 3.32-4.10) for tramadol, 4.50 (95% CI, 4.23-4.79) for oxycodone, 4.75 (95% CI, 4.26-5.29) for methadone, 5.11 (95% CI, 4.73-5.53) for hydrocodone, 6.71 (95% CI, 6.29-7.16) for fentanyl, and 9.93 (95% CI, 9.31-10.59) for heroin.

Table 2 displays factors associated with buprenorphine positivity from the multivariate logistic regression. Compared with patients receiving care at substance use disorder treatment centers, buprenorphine positivity was significantly more likely at behavioral health practices (aOR, 1.30; 95% CI, 1.24-1.38). Compared with patients aged 18 to 24 years, buprenorphine positivity was higher in all other age groups, particularly those aged 35 to 44 years (aOR, 1.94; 95% CI, 1.81-2.07). Compared with patients with private insurance, positivity rates were slightly higher for patients with Medicaid (aOR, 1.06; 95% CI, 1.01-1.11), but not statistically different for other payer groups. Positivity rates were highest in the Mid-Atlantic and East South Central Census divisions and lowest in the Pacific division.

**Table 2.** Logistic Regression Results for Buprenorphine Detection^a^

Characteristic	Probability^b^	Adjusted odds ratio^c^	*P* value^c^
Sex			
Female	0.859 (0.853-0.864)	1 [Reference]	NA
Male	0.844 (0.839-0.849)	0.89 (0.86-0.93)	<.001
Age, y			
18-24	0.786 (0.772-0.799)	1 [Reference]	NA
25-34	0.865 (0.859-0.871)	1.75 (1.64-1.86)	<.001
35-44	0.877 (0.870-0.883)	1.94 (1.81-2.07)	<.001
45-54	0.862 (0.854-0.871)	1.71 (1.59-1.85)	<.001
≥55	0.856 (0.845-0.866)	1.62 (1.49-1.76)	<.001
US Census Division			
East North Central	0.819 (0.810-0.827)	1 [Reference]	NA
East South Central	0.882 (0.871-0.892)	1.65 (1.54-1.79)	<.001
Mid-Atlantic	0.884 (0.875-0.893)	1.69 (1.58-1.82)	<.001
Mountain	0.835 (0.821-0.849)	1.12 (1.04-1.21)	.003
New England	0.881 (0.869-0.892)	1.64 (1.51-1.78)	<.001
Pacific	0.804 (0.792-0.816)	0.91 (0.86-0.97)	.002
South Atlantic	0.822 (0.813-0.832)	1.03 (0.97-1.08)	.36
West North Central	0.881 (0.861-0.899)	1.64 (1.44-1.88)	<.001
West South Central	0.830 (0.807-0.852)	1.08 (0.97-1.22)	.17
Health care practice specialty			
Substance use disorder treatment	0.836 (0.830-0.842)	1 [Reference]	NA
Behavioral health	0.870 (0.862-0.876)	1.30 (1.24-1.38)	<.001
Primary care physician	0.847 (0.840-0.853)	1.08 (1.04-1.13)	<.001
Payer group			
Private insurance	0.847 (0.840-0.854)	1 [Reference]	NA
Medicaid	0.854 (0.848-0.860)	1.06 (1.01-1.11)	.02
Medicare	0.852 (0.840-0.862)	1.04 (0.96-1.12)	.35
Uninsured	0.853 (0.844-0.862)	1.05 (0.99-1.12)	.11
Collection year			
2015	0.850 (0.843-0.857)	1 [Reference]	NA
2016	0.843 (0.835-0.851)	0.95 (0.90-1.00)	.06
2017	0.851 (0.842-0.860)	1.01 (0.95-1.07)	.73
2018	0.861 (0.853-0.869)	1.10 (1.04-1.16)	.001
2019	0.851 (0.842-0.859)	1.01 (0.95-1.07)	.83

^a^ A logistic regression model of buprenorphine detection was performed containing collection year, US Census division, sex, age, health care specialty, and payer group. The total model fit was significant (χ^2^ = 1225.8; *P* < .001).

^b^ Marginal probability predictions (least square mean) and Sidak-corrected confidence intervals were estimated.

^c^ Adjusted odds ratios and *P* values were estimated for each covariate relative to the reference level.

Table 3 displays factors associated with positivity for other substances. For most of the substances examined, the positivity rate was highest for patients at substance use disorder treatment centers. Alcohol positivity was higher in primary care practices (aOR, 1.20; 95% CI, 1.12-1.28), and similar patterns held for marijuana positivity. Odds of positivity by age differed by substances. For example, younger individuals were consistently more likely to have positivity for fentanyl and heroin. Comparing those aged 55 years and older with those aged 18 to 24 years, the aOR for fentanyl positivity was 0.46 (95% CI, 0.39-0.54) and for heroin positivity, 0.35 (95% CI, 0.29-0.42). By contrast, older individuals were more likely than younger individuals to be positive for benzodiazepines, alcohol, gabapentin, tramadol, and oxycodone. For example, the aOR of oxycodone positivity was 1.42 (95% CI, 1.22-1.64) for individuals aged 55 years or older compared with those aged 18 to 24 years. Men had higher, or similar, positivity rates as women for every substance except for gabapentin (aOR, 0.72; 95% CI, 0.67-0.78), tramadol (aOR, 0.84; 95% CI, 0.76-0.93), benzodiazepines (aOR, 0.83; 95% CI, 0.80-0.87), hydrocodone (aOR, 0.72; 95% CI, 0.67-0.78), and oxycodone (aOR, 0.84; 95% CI, 0.79-0.89). For example, the aOR for fentanyl among men was 1.13 (95% CI, 1.04-1.19).

**Table 3.** Adjusted Odds Ratios for Nonprescribed Substances

Characteristic	Adjusted odds ratios (95% CI)^a^											
	Marijuana	Alcohol	Cocaine	Methamphetamine	Heroin	Fentanyl	Methadone	Hydrocodone	Oxycodone	Tramadol	Benzodiazepines	Gabapentin
Sex												
Female	1 [Reference]	1 [Reference]	1 [Reference]	1 [Reference]	1 [Reference]	1 [Reference]	1 [Reference]	1 [Reference]	1 [Reference]	1 [Reference]	1 [Reference]	1 [Reference]
Male	1.53 (1.47-1.58)	1.29 (1.22-1.35)	1.13 (1.07-1.18)	1.04 (0.99-1.10)	1.11 (1.04-1.19)	1.13 (1.06-1.21)	0.91 (0.82-1.01)	0.72 (0.67-0.78)	0.84 (0.79-0.89)	0.84 (0.76-0.93)	0.83 (0.80-0.87)	0.72 (0.67-0.78)
Age, y												
18-24	1 [Reference]	1 [Reference]	1 [Reference]	1 [Reference]	1 [Reference]	1 [Reference]	1 [Reference]	1 [Reference]	1 [Reference]	1 [Reference]	1 [Reference]	1 [Reference]
25-34	0.59 (0.56-0.63)	1.27 (1.15-1.41)	0.82 (0.76-0.89)	0.99 (0.90-1.09)	0.81 (0.73-0.90)	0.77 (0.69-0.85)	1.23 (1.00-1.52)	1.15 (0.99-1.34)	1.17 (1.04-1.31)	1.34 (1.07-1.69)	0.99 (0.92-1.07)	0.96 (0.82-1.13)
35-44	0.47 (0.44-0.51)	1.43 (1.28-1.59)	0.72 (0.66-0.78)	0.96 (0.87-1.06)	0.59 (0.53-0.66)	0.56 (0.50-0.63)	1.44 (1.17-1.80)	1.48 (1.27-1.73)	1.27 (1.13-1.43)	1.59 (1.27-2.01)	1.07 (0.98-1.15)	1.19 (1.02-1.41)
45-54	0.35 (0.33-0.38)	1.69 (1.51-1.90)	0.70 (0.63-0.77)	0.72 (0.64-0.82)	0.51 (0.44-0.58)	0.50 (0.43-0.57)	1.43 (1.13-1.82)	1.67 (1.42-1.98)	1.33 (1.16-1.51)	1.88 (1.48-2.42)	1.16 (1.07-1.27)	1.43 (1.20-1.71)
≥55	0.28 (0.26-0.31)	1.81 (1.60-2.06)	0.49 (0.43-0.55)	0.47 (0.41-0.55)	0.35 (0.29-0.42)	0.46 (0.39-0.54)	1.38 (1.05-1.81)	1.82 (1.52-2.19)	1.42 (1.22-1.64)	1.83 (1.39-2.41)	1.23 (1.12-1.36)	1.40 (1.15-1.70)
US Census division												
East North Central	1 [Reference]	1 [Reference]	1 [Reference]	1 [Reference]	1 [Reference]	1 [Reference]	1 [Reference]	1 [Reference]	1 [Reference]	1 [Reference]	1 [Reference]	1 [Reference]
East South Central	0.70 (0.66-0.75)	0.95 (0.85-1.06)	0.33 (0.29-0.37)	2.11 (1.92-2.31)	0.26 (0.20-0.32)	0.38 (0.33-0.44)	1.20 (0.95-1.50)	2.40 (2.13-2.71)	1.04 (0.93-1.17)	0.61 (0.49-0.76)	1.25 (1.17-1.34)	1.18 (1.06-1.32)
Mid-Atlantic	0.89 (0.83-0.95)	1.04 (0.94-1.15)	0.84 (0.77-0.91)	0.67 (0.58-0.77)	0.97 (0.86-1.08)	0.65 (0.58-0.74)	1.41 (1.14-1.72)	0.88 (0.75-1.01)	1.01 (0.91-1.12)	0.69 (0.57-0.84)	1.29 (1.20-1.38)	0.96 (0.83-1.09)
Mountain	0.92 (0.85-0.99)	1.15 (1.04-1.27)	0.40 (0.35-0.45)	2.22 (2.01-2.45)	1.39 (1.23-1.58)	0.16 (0.13-0.19)	1.35 (1.05-1.71)	1.09 (0.91-1.29)	0.94 (0.82-1.08)	0.74 (0.59-0.92)	1.16 (1.07-1.27)	0.89 (0.75-1.04)
New England	1.84 (1.72-1.97)	1.64 (1.50-1.79)	1.05 (0.96-1.14)	0.52 (0.44-0.62)	0.50 (0.42-0.59)	1.19 (1.07-1.33)	2.30 (1.87-2.82)	0.40 (0.31-0.51)	0.86 (0.75-0.97)	0.39 (0.28-0.51)	1.21 (1.12-1.31)	0.90 (0.77-1.05)
Pacific	1.24 (1.17-1.32)	1.27 (1.17-1.38)	0.30 (0.27-0.33)	4.60 (4.27-4.95)	2.67 (2.44-2.93)	0.15 (0.13-0.17)	1.66 (1.36-2.02)	1.30 (1.14-1.49)	0.69 (0.61-0.78)	0.54 (0.44-0.66)	0.99 (0.92-1.07)	0.53 (0.43-0.64)
South Atlantic	1.05 (1.00-1.11)	1.34 (1.24-1.45)	1.13 (1.06-1.21)	1.24 (1.13-1.35)	0.95 (0.86-1.05)	0.76 (0.69-0.83)	2.27 (1.94-2.65)	1.10 (0.98-1.24)	1.45 (1.33-1.58)	0.78 (0.67-0.90)	1.32 (1.24-1.40)	0.81 (0.71-0.92)
West North Central	0.89 (0.80-0.98)	0.80 (0.68-0.93)	0.15 (0.11-0.20)	2.62 (2.30-2.98)	0.74 (0.57-0.94)	0.58 (0.47-0.71)	1.90 (1.36-2.61)	1.19 (0.91-1.54)	0.93 (0.74-1.15)	1.06 (0.78-1.41)	1.24 (1.08-1.41)	0.95 (0.73-1.23)
West South Central	0.75 (0.66-0.84)	1.32 (1.11-1.57)	0.29 (0.23-0.36)	2.46 (2.08-2.90)	0.47 (0.34-0.63)	0.11 (0.07-0.18)	0.80 (0.48-1.25)	2.41 (2.00-2.89)	0.62 (0.50-0.77)	0.92 (0.66-1.24)	1.43 (1.26-1.62)	0.71 (0.53-0.94)
Health care practice specialty												
Substance use treatment	1 [Reference]	1 [Reference]	1 [Reference]	1 [Reference]	1 [Reference]	1 [Reference]	1 [Reference]	1 [Reference]	1 [Reference]	1 [Reference]	1 [Reference]	1 [Reference]
Behavioral health	1.03 (0.98-1.08)	1.05 (0.99-1.12)	0.74 (0.69-0.80)	0.85 (0.79-0.91)	0.80 (0.72-0.87)	0.64 (0.59-0.71)	1.18 (1.02-1.36)	0.76 (0.71-0.82)	0.85 (0.78-0.92)	0.96 (0.82-1.11)	0.95 (0.90-1.00)	1.26 (1.15-1.39)
Primary care	1.10 (1.05-1.15)	1.20 (1.12-1.28)	0.84 (0.79-0.89)	0.82 (0.77-0.87)	0.95 (0.88-1.03)	0.56 (0.51-0.61)	1.08 (0.95-1.22)	0.95 (0.90-1.00)	1.01 (0.94-1.08)	0.85 (0.73-0.98)	0.96 (0.92-1.01)	0.79 (0.72-0.87)
Payer group												
Private insurance	1 [Reference]	1 [Reference]	1 [Reference]	1 [Reference]	1 [Reference]	1 [Reference]	1 [Reference]	1 [Reference]	1 [Reference]	1 [Reference]	1 [Reference]	1 [Reference]
Medicaid	1.33 (1.27-1.39)	0.67 (0.63-0.71)	1.31 (1.23-1.39)	1.95 (1.82-2.10)	1.42 (1.31-1.54)	1.20 (1.11-1.31)	1.28 (1.12-1.47)	0.74 (0.67-0.81)	0.78 (0.73-0.84)	1.20 (1.05-1.38)	0.96 (0.91-1.00)	1.94 (1.74-2.17)
Medicare	1.29 (1.20-1.40)	0.65 (0.58-0.72)	1.16 (1.05-1.29)	1.67 (1.48-1.88)	1.01 (0.86-1.18)	0.99 (0.85-1.14)	1.16 (0.92-1.44)	0.96 (0.83-1.11)	0.86 (0.76-0.97)	1.24 (1.00-1.53)	1.29 (1.19-1.40)	2.07 (1.78-2.41)
Uninsured	1.22 (1.15-1.30)	0.98 (0.90-1.06)	1.10 (1.01-1.19)	1.79 (1.63-1.97)	0.96 (0.85-1.08)	0.94 (0.84-1.05)	1.09 (0.91-1.30)	1.14 (1.02-1.28)	0.97 (0.88-1.06)	1.25 (1.05-1.50)	1.03 (0.97-1.10)	0.91 (0.77-1.07)
Collection year												
2015	1 [Reference]	1 [Reference]	1 [Reference]	1 [Reference]	1 [Reference]	1 [Reference]	1 [Reference]	1 [Reference]	1 [Reference]	1 [Reference]	1 [Reference]	1 [Reference]
2016	1.13 (1.07-1.19)	1.05 (0.98-1.14)	1.11 (1.04-1.19)	1.70 (1.55-1.88)	0.93 (0.85-1.02)	1.79 (1.60-1.99)	1.04 (0.90-1.20)	1.09 (0.99-1.21)	1.08 (1.00-1.17)	1.01 (0.87-1.18)	1.04 (0.99-1.10)	1.10 (0.95-1.27)
2017	1.18 (1.12-1.25)	0.99 (0.91-1.07)	1.38 (1.28-1.49)	2.18 (1.98-2.40)	0.71 (0.64-0.79)	2.39 (2.13-2.68)	0.89 (0.75-1.06)	0.97 (0.86-1.09)	0.91 (0.82-1.00)	1.22 (1.04-1.43)	0.86 (0.81-0.92)	1.06 (0.92-1.21)
2018	1.37 (1.30-1.44)	1.00 (0.92-1.07)	1.14 (1.06-1.22)	2.75 (2.52-2.99)	0.72 (0.65-0.80)	2.75 (2.47-3.06)	0.82 (0.70-0.97)	0.71 (0.63-0.80)	0.63 (0.57-0.69)	0.97 (0.83-1.14)	0.70 (0.65-0.74)	1.16 (1.03-1.31)
2019	1.45 (1.37-1.53)	0.98 (0.91-1.06)	1.18 (1.09-1.27)	3.60 (3.31-3.91)	0.76 (0.69-0.84)	4.02 (3.63-4.45)	0.74 (0.62-0.88)	0.55 (0.48-0.62)	0.63 (0.57-0.70)	0.69 (0.58-0.82)	0.67 (0.63-0.71)	1.21 (1.08-1.36)
Buprenorphine detection status												
Positive	1 [Reference]	1 [Reference]	1 [Reference]	1 [Reference]	1 [Reference]	1 [Reference]	1 [Reference]	1 [Reference]	1 [Reference]	1 [Reference]	1 [Reference]	1 [Reference]
Negative	1.33 (1.27-1.39)	1.24 (1.16-1.32)	2.64 (2.50-2.78)	2.14 (2.01-2.27)	9.93 (9.31-10.59)	6.71 (6.29-7.16)	4.75 (4.26-5.29)	5.11 (4.73-5.53)	4.50 (4.23-4.79)	3.69 (3.32-4.10)	1.48 (1.41-1.56)	1.05 (0.94-1.15)

^a^ Adjusted odds ratios relative to reference level and 95% confidence intervals were estimated.

There were significant differences by payer. Compared with those with private insurance, those with Medicaid had significantly higher positivity rates for most substances (eg, fentanyl: OR, 1.20; 95% CI, 1.11-1.31) but significantly lower rates for alcohol (OR, 0.67; 95% CI, 0.63-0.71), hydrocodone (OR, 0.74; 95% CI, 0.67-0.81), and oxycodone (OR, 0.78; 95% CI, 0.73-0.84). Patients with Medicare had significantly higher positivity rates than patients with private insurance for substances such as gabapentin (OR, 2.07; 95% CI, 1.78-2.41) but significantly lower rates for alcohol (OR, 0.65; 95% CI, 0.58-0.72) and oxycodone (OR, 0.86; 95% CI, 0.76-0.97). Compared with patients with private insurance, individuals without insurance had higher or similar odds of positivity for every substance. Geographic differences were compared across the Census divisions with the East North Central as the reference group. The Pacific region had the highest positivity for heroin (OR, 2.67; 95% CI, 2.44-2.93) and methamphetamine (OR, 4.60; 95% CI, 4.27-4.95) relative to the reference group. New England had the highest positivity for alcohol (OR, 1.64; 95% CI, 1.50-1.79) and fentanyl (OR, 1.19; 95% CI, 1.07-1.33), and South Atlantic had the highest positivity for cocaine (OR, 1.13; 95% CI, 1.06-1.21) and oxycodone (OR, 1.45, 95% CI, 1.33-1.58). Patients whose specimens were positive for buprenorphine were significantly less likely to be positive for other opioids (eg, fentanyl: OR for buprenorphine-negative samples, 6.71; 95% CI, 6.29-7.16; heroin: OR for buprenorphine-negative samples, 9.93; 95% CI, 9.31-10.59).

Our sensitivity analysis examining second specimens found higher buprenorphine positivity rates than first specimens (127 469 of 137 722 [92.6%] vs 128 240 of 150 000 [85.5%]). However, similar patterns of copositivity with other substances as first specimens were observed (eTable 3 and eTable 4 in the Supplement).

## Discussion

Drawing on a unique database of UDTs, this study examined trends in drug positivity among patients prescribed buprenorphine from 2013 to 2019. Several key findings emerge from the study. First, more than half of patients (52.18%) showed positivity for nonprescribed substances, particularly for marijuana, benzodiazepines, and gabapentin. Second, overall positivity rates fluctuated during the study period. For example, they increased substantially for methamphetamine and fentanyl but not to the same extent for cocaine and heroin. Third, buprenorphine positivity was consistently associated with lower positivity of all other substances except for gabapentin. The OR was largest for heroin. Fourth, patient age and sex, setting of care, and geographic region were associated with drug positivity among patients prescribed buprenorphine, but the results varied by substance. For example, older patients, women, those with private insurance, and those being treated in primary care were more likely to have oxycodone positivity, whereas fentanyl positivity was highest among young men, those with Medicaid, those being treated at substance use treatment centers, and those living in New England.

The study population is based on the first sample provided by each patient to Millennium Health. We found that 14.5% of patients were buprenorphine negative when they provided their specimen. Buprenorphine negativity may indicate that patients were not taking their prescribed medication. However, it is unclear whether all patients had received their first prescribed buprenorphine dose at the time of the first tested UDT. As noted, our sensitivity analysis found similar patterns with second specimens. Furthermore, it is notable that buprenorphine positivity was most associated with lower positivity for other opioids. This is expected, given that buprenorphine reduces cravings for these opioids and also has a ceiling effect on their euphoric potential.^18^ Our findings further indicate that patients often have complex polysubstance use, with specific use patterns varying across populations and geographic areas. Future studies of polysubstance use could examine issues such as factors associated with being positive for multiple types of nonprescribed substances or multiple pharmacological categories.

Our findings add nuance to epidemiological efforts that have been mounted in recent years to track the complex patterns of substance use among patients with OUD.^19^ For example, we were able to find granular variation in risk factors across demographic groups and settings of care. This knowledge could help public health agencies to direct alerts about drug use trends and to provide targeted risk reduction tools (eg, harm reduction supplies). These data can also provide value for efforts focused on geographic regions and specific payers (eg, Medicaid programs or commercial insurance). Study data also augment what can be learned through self-reported substance use measures. While self-reports are often accurate, patients in some situations may not fully disclose their recent substance use histories due to social desirability bias or recall bias. This self-report bias is likely to be largest at the beginning of treatment.^17^ Furthermore, given the substantial adulteration of the drug supply with fentanyl, some patients may lack awareness of the specific substances they have used.

### Limitations

This study has limitations. First, as noted, first specimens may include individuals who have not yet received their first dose of buprenorphine. Further research is needed to identify the longitudinal associations between buprenorphine adherence and illicit substance use. Second, not all patients were tested for all analytes, which may introduce some selection bias. However, with the exception of gabapentin (added in 2014), the analytes we examined were tested for between 77 299 patients (51.53%) for tramadol and 136 187 patients (90.79%) for oxycodone, making them common in clinical practice. Furthermore, not all psychoactive substances (eg, synthetic cannabinoids, cathinones, and fentanyl analogues) were included in this study. Third, patients included in the analysis may have had an incomplete or inaccurate medication list from the ordering clinician, which would result in possible inclusion of positive results associated with an unreported prescription medication. Fourth, testing data cannot reveal use of substances that occurs outside of the window of detection (typically 1-3 days for most analytes evaluated). Fifth, buprenorphine is sometimes used as a medication for management of chronic pain.^20^ To account for this limitation, we excluded patients from pain management clinics and formulations of buprenorphine that are indicated only for the treatment of pain. Sixth, some patients may first be screened with a presumptive (eg, in-office) test performed with immunoassay technology; study findings may not generalize to patients that only receive presumptive tests. Additionally, the data set ended in 2019, before the emergence of the COVID-19 pandemic in the United States, which altered the delivery of OUD treatment.^21^

## Conclusions

This study highlights the utility of UDT data in public health surveillance efforts related to patients treated with buprenorphine for OUD. Amid national efforts to expand buprenorphine treatment, updating these analyses can help to identify opportunities for interventions and insights into the rapidly evolving patterns of substance use among this patient population.

## Notes

Supplement. **eTable 1. **Characteristics of UDT Specimens Tested for Prescribed Buprenorphine Stratified by Collection Year **eTable 2. **Crude Positivity Rates Stratified by Buprenorphine Detection and Collection Year for Nonprescribed and/or Illicit Drugs **eTable 3. **Comparison of Characteristics for UDT Specimens From the First and Second Specimen Sample Populations Collected Between January 1, 2013, and December 31, 2019 **eTable 4. **Comparison of Logistic Regression Results for UDT Specimens From the First and Second Specimen Sample Populations **eFigure. **Positivity Rates for All Substances Stratified by Year and Patient Positivity for Buprenorphine
