# Error in Discussion

**DOI:** 10.1001/jamanetworkopen.2021.33270

**Published:** 2021-10-01

In the Original Investigation titled “Polysubstance Use Among Patients Treated With Buprenorphine From a National Urine Drug Test Database,”^1^ published September 10, 2021, there was an error in the Discussion. The third sentence of the first paragraph should have read, “First, nearly half of patients (47.58%) showed positivity for nonprescribed substances, particularly for marijuana, benzodiazepines, and gabapentin.” This article has been corrected.^1^
