# Supplementary material for: Polysubstance Use Among Patients Treated With Buprenorphine From a National Urine Drug Test Database
Source: JAMA Netw Open. 2021 Sep 10;4(9):e2123019. doi: 10.1001/jamanetworkopen.2021.23019 (PMC8433604; doi:10.1001/jamanetworkopen.2021.23019)
Supplement: Supplement. — eTable 1. Characteristics of UDT Specimens Tested for Prescribed Buprenorphine Stratified by Collection Year eTable 2. Crude Positivity Rates Stratified by Buprenorphine Detection and Collection Year for Nonprescribed and/or Illicit Drugs eTable 3. Comparison of Characteristics for UDT Specimens From the First and Second Specimen Sample Populations Collected Between January 1, 2013, and December 31, 2019 eTable 4. Comparison of Logistic Regression Results for UDT Specimens From the First and Second Specimen Sample Populations eFigure. Positivity Rates for All Substances Stratified by Year and Patient Positivity for Buprenorphine [file jamanetwopen-e2123019-s001.pdf]

Supplemental Online Content

Saloner B, Whitley P, LaRue L, Dawson E, Huskey A. Polysubstance use among patients treated with buprenorphine from a national urine drug test database. *JAMA Netw Open.* 2021;4(9):e2123019. doi:10.1001/jamanetworkopen.2021.23019

eTable 1. Characteristics of UDT Specimens Tested for Prescribed Buprenorphine Stratified by Collection Year

eTable 2. Crude Positivity Rates Stratified by Buprenorphine Detection and Collection Year for Nonprescribed and/or Illicit Drugs

eTable 3. Comparison of Characteristics for UDT Specimens From the First and Second Specimen Sample Populations Collected Between January 1, 2013, and December 31, 2019

eTable 4. Comparison of Logistic Regression Results for UDT Specimens From the First and Second Specimen Sample Populations

eFigure. Positivity Rates for All Substances Stratified by Year and Patient Positivity for Buprenorphine

This supplemental material has been provided by the authors to give readers additional information about their work.

© 2021 Saloner B et al. *JAMA Network Open.*

eTable 1. Characteristics of UDT Specimens Tested for Prescribed Buprenorphine Stratified by Collection Year

	Specimens Tested, No. (%)							
**Characteristics**	**2013**	**2014**	**2015**	**2016**	**2017**	**2018**	**2019**	**Total**
Unique Patient Specimens	36,523 (100.00%)	24,792 (100.00%)	25,914 (100.00%)	17,379 (100.00%)	12,828 (100.00%)	16,583 (100.00%)	15,981 (100.00%)	150,000 (100.00%)
Sex								
F	15,755 (43.14%)	10,926 (44.07%)	11,568 (44.64%)	7,882 (45.35%)	6,091 (47.48%)	8,019 (48.36%)	7,652 (47.88%)	67,893 (45.26%)
M	20,768 (56.86%)	13,866 (55.93%)	14,346 (55.36%)	9,497 (54.65%)	6,737 (52.52%)	8,564 (51.64%)	8,329 (52.12%)	82,107 (54.74%)
Age, y								
18-24	4,820 (13.20%)	3,092 (12.47%)	2,821 (10.89%)	1,490 (8.57%)	978 (7.62%)	1,030 (6.21%)	920 (5.76%)	15,151 (10.10%)
25-34	15,198 (41.61%)	10,617 (42.82%)	10,913 (42.11%)	7,250 (41.72%)	5,038 (39.27%)	6,687 (40.32%)	6,446 (40.34%)	62,149 (41.43%)
35-44	8,867 (24.28%)	6,154 (24.82%)	6,741 (26.01%)	4,909 (28.25%)	3,822 (29.79%)	5,101 (30.76%)	5,087 (31.83%)	40,681 (27.12%)
45-54	5,038 (13.79%)	3,185 (12.85%)	3,499 (13.50%)	2,329 (13.40%)	1,805 (14.07%)	2,264 (13.65%)	2,066 (12.93%)	20,186 (13.46%)
55+	2,519 (6.90%)	1,687 (6.80%)	1,901 (7.34%)	1,380 (7.94%)	1,177 (9.18%)	1,482 (8.94%)	1,447 (9.05%)	11,593 (7.73%)
US Census Division								
East North Central	8,207 (22.47%)	6,504 (26.23%)	6,728 (25.96%)	5,570 (32.05%)	3,364 (26.22%)	4,785 (28.85%)	4,875 (30.50%)	40,033 (26.69%)
East South Central	2,168 (5.94%)	1,696 (6.84%)	2,387 (9.21%)	1,670 (9.61%)	1,402 (10.93%)	1,739 (10.49%)	1,421 (8.89%)	12,483 (8.32%)
Mid Atlantic	9,283 (25.42%)	5,446 (21.97%)	5,337 (20.60%)	2,459 (14.15%)	949 (7.40%)	1,249 (7.53%)	771 (4.82%)	25,494 (17.00%)
Mountain	1,700 (4.65%)	1,503 (6.06%)	1,264 (4.88%)	966 (5.56%)	1,255 (9.78%)	1,327 (8.00%)	1,512 (9.46%)	9,527 (6.35%)
New England	1,936 (5.30%)	931 (3.76%)	2,327 (8.98%)	1,598 (9.20%)	1,165 (9.08%)	961 (5.80%)	748 (4.68%)	9,666 (6.44%)
Pacific	1,342 (3.67%)	1,358 (5.48%)	1,465 (5.65%)	1,295 (7.45%)	1,255 (9.78%)	2,918 (17.60%)	3,751 (23.47%)	13,384 (8.92%)
South Atlantic	9,109 (24.94%)	5,874 (23.69%)	5,338 (20.60%)	2,860 (16.46%)	2,552 (19.89%)	2,721 (16.41%)	1,944 (12.16%)	30,398 (20.27%)
West North Central	460 (1.26%)	193 (0.78%)	312 (1.20%)	275 (1.58%)	399 (3.11%)	675 (4.07%)	801 (5.01%)	3,115 (2.08%)
West South Central	2,318 (6.35%)	1,287 (5.19%)	756 (2.92%)	686 (3.95%)	487 (3.80%)	208 (1.25%)	158 (0.99%)	5,900 (3.93%)
Health Care Practice Specialty								
Behavioral Health	3,702 (10.14%)	2,750 (11.09%)	4,447 (17.16%)	3,305 (19.02%)	2,463 (19.20%)	2,918 (17.60%)	2,990 (18.71%)	22,575 (15.05%)
Primary Care Physician	12,279 (33.62%)	7,413 (29.90%)	7,069 (27.28%)	5,321 (30.62%)	4,498 (35.06%)	4,685 (28.25%)	3,864 (24.18%)	45,129 (30.09%)
Substance Use Treatment	20,542 (56.24%)	14,629 (59.01%)	14,398 (55.56%)	8,753 (50.37%)	5,867 (45.74%)	8,980 (54.15%)	9,127 (57.11%)	82,296 (54.86%)
Payor Group								
Medicaid	0 (0.00%)	0 (0.00%)	10,729 (41.40%)	8,150 (46.90%)	6,144 (47.90%)	9,083 (54.77%)	10,114 (63.29%)	44,220 (29.48%)
Medicare	0 (0.00%)	0 (0.00%)	2,053 (7.92%)	1,397 (8.04%)	1,181 (9.21%)	1,422 (8.58%)	1,226 (7.67%)	7,279 (4.85%)

© 2021 Saloner B et al. *JAMA Network Open.*

Private insurance	0 (0.00%)	0 (0.00%)	8,865 (34.21%)	5,112 (29.41%)	3,451 (26.90%)	3,316 (20.00%)	2,981 (18.65%)	23,725 (15.82%)
Uninsured	0 (0.00%)	0 (0.00%)	4,201 (16.21%)	2,591 (14.91%)	1,748 (13.63%)	2,295 (13.84%)	1,560 (9.76%)	12,395 (8.26%)
Unknown	36,523 (100.00%)	24,792 (100.00%)	66 (0.25%)	129 (0.74%)	304 (2.37%)	467 (2.82%)	100 (0.63%)	62,381 (41.59%)
UDT Positives, No. (% positivity)[a]								
Buprenorphine	32,076 (87.82%)	21,420 (86.40%)	21,853 (84.33%)	14,524 (83.57%)	10,852 (84.60%)	14,124 (85.17%)	13,391 (83.79%)	128,240 (85.49%)
Benzodiazepines	5,805 (19.80%)	4,329 (21.25%)	4,384 (20.24%)	2,699 (20.72%)	1,631 (17.69%)	1,898 (14.58%)	1,738 (13.87%)	22,484 (18.87%)
Cocaine	2,005 (6.08%)	1,872 (8.19%)	2,416 (9.91%)	1,657 (10.78%)	1,300 (11.52%)	1,431 (9.43%)	1,356 (9.95%)	12,037 (8.87%)
Alcohol	2,818 (15.05%)	1,924 (14.40%)	2,102 (15.08%)	1,330 (15.56%)	1,134 (15.16%)	1,446 (14.09%)	1,461 (13.30%)	12,215 (14.67%)
Fentanyl	131 (0.61%)	242 (1.66%)	685 (3.91%)	911 (6.63%)	728 (7.03%)	1,056 (7.38%)	1,464 (10.77%)	5,217 (4.94%)
Gabapentin[b]	0 (NA)	143 (8.79%)	526 (13.36%)	397 (14.40%)	497 (13.47%)	1,093 (15.46%)	1,117 (16.77%)	3,773 (14.66%)
Heroin	986 (3.47%)	1,025 (5.01%)	1,428 (6.53%)	976 (6.43%)	559 (4.99%)	859 (5.73%)	952 (6.85%)	6,785 (5.38%)
Hydrocodone	1,652 (4.89%)	1,105 (4.78%)	998 (4.06%)	694 (4.63%)	459 (4.18%)	458 (3.11%)	333 (2.39%)	5,699 (4.19%)
Methadone	743 (2.30%)	482 (2.31%)	459 (2.03%)	323 (2.20%)	209 (1.94%)	242 (1.69%)	209 (1.58%)	2,667 (2.07%)
Methamphetamine	504 (1.53%)	553 (2.47%)	933 (4.14%)	926 (7.57%)	1,054 (10.71%)	2,107 (15.07%)	2,663 (19.45%)	8,740 (6.85%)
Marijuana	1,149 (23.89%)	4,277 (23.17%)	4,876 (24.91%)	3,458 (27.35%)	2,839 (27.48%)	3,976 (30.57%)	3,989 (32.13%)	24,564 (26.92%)
Oxycodone	2,189 (6.54%)	1,582 (6.92%)	1,715 (7.08%)	1,156 (7.54%)	706 (6.24%)	646 (4.27%)	592 (4.28%)	8,586 (6.30%)
Tramadol	98 (3.00%)	334 (2.68%)	384 (2.54%)	319 (2.59%)	285 (3.01%)	307 (2.41%)	226 (1.89%)	1,953 (2.53%)

Abbreviations: Urine Drug Testing (UDT)

Notes:

150,000 randomly selected patient specimens collected between 2013 and 2019 and tested for prescribed buprenorphine were evaluated.

[a] All positive numbers and positivity rates are based on the non-prescribed analyte population except buprenorphine

[b] No gabapentin UDT results occurred prior to June 2014.

© 2021 Saloner B et al. *JAMA Network Open.*

eTable 2. Crude Positivity Rates (%, 95% CI) Stratified by Buprenorphine Detection and Collection Year for Nonprescribed and/or Illicit Drugs

Analyte	Buprenorphine Detection	2013	2014	2015	2016	2017	2018	2019
Alcohol	negative	16.61 [15.19-18.11]	16.29 [14.63-18.05]	18.27 [16.68-19.95]	17.28 [15.41-19.27]	17.29 [15.17-19.56]	16.53 [14.72-18.47]	15.27 [13.62-17.04]
Alcohol	positive	14.80 [14.26-15.36]	14.10 [13.47-14.75]	14.48 [13.85-15.13]	15.19 [14.36-16.05]	14.76 [13.89-15.66]	13.65 [12.94-14.39]	12.93 [12.25-13.63]
Alcohol	total	15.05 [14.54-15.57]	14.40 [13.81-15.01]	15.08 [14.49-15.68]	15.56 [14.80-16.35]	15.16 [14.35-15.99]	14.09 [13.42-14.78]	13.30 [12.67-13.95]
Benzodiazepines	negative	25.78 [24.39-27.20]	27.59 [25.96-29.27]	27.59 [26.07-29.14]	25.59 [23.79-27.45]	22.83 [20.70-25.06]	18.21 [16.52-19.99]	15.60 [14.07-17.23]
Benzodiazepines	positive	18.92 [18.44-19.40]	20.21 [19.62-20.82]	18.92 [18.35-19.49]	19.71 [18.97-20.48]	16.71 [15.89-17.56]	13.93 [13.29-14.59]	13.52 [12.87-14.19]
Benzodiazepines	total	19.80 [19.34-20.26]	21.25 [20.69-21.82]	20.24 [19.71-20.78]	20.72 [20.03-21.43]	17.69 [16.91-18.48]	14.58 [13.97-15.19]	13.87 [13.27-14.48]
Cocaine	negative	10.93 [9.98-11.94]	14.14 [12.95-15.40]	19.01 [17.79-20.28]	21.01 [19.49-22.60]	20.48 [18.65-22.42]	18.32 [16.76-19.97]	20.30 [18.68-22.01]
Cocaine	positive	5.41 [5.15-5.67]	7.23 [6.87-7.60]	8.17 [7.80-8.55]	8.62 [8.13-9.12]	9.80 [9.21-10.41]	7.85 [7.39-8.33]	7.85 [7.36-8.36]
Cocaine	total	6.08 [5.82-6.34]	8.19 [7.83-8.55]	9.91 [9.54-10.29]	10.78 [10.29-11.28]	11.52 [10.93-12.12]	9.43 [8.97-9.91]	9.95 [9.45-10.47]
Fentanyl	negative	2.02 [1.54-2.60]	4.89 [4.00-5.90]	12.49 [11.28-13.77]	20.45 [18.86-22.11]	21.52 [19.56-23.59]	22.14 [20.40-23.97]	32.46 [30.54-34.42]
Fentanyl	positive	0.39 [0.30-0.49]	1.12 [0.94-1.32]	2.28 [2.04-2.53]	3.66 [3.32-4.02]	4.30 [3.88-4.75]	4.79 [4.41-5.18]	6.38 [5.93-6.84]
Fentanyl	total	0.61 [0.51-0.73]	1.66 [1.45-1.88]	3.91 [3.62-4.20]	6.63 [6.22-7.06]	7.03 [6.54-7.54]	7.38 [6.95-7.82]	10.77 [10.25-11.30]
Gabapentin	negative	NA	10.38 [7.25-14.26]	14.03 [11.69-16.65]	11.28 [8.10-15.15]	14.45 [11.29-18.11]	15.54 [13.26-18.05]	17.55 [15.44-19.82]
Gabapentin	positive	NA	8.41 [6.96-10.05]	13.20 [12.03-14.43]	14.84 [13.45-16.32]	13.34 [12.19-14.56]	15.44 [14.55-16.37]	16.60 [15.62-17.61]
Gabapentin	total	NA	8.79 [7.46-10.28]	13.36 [12.32-14.47]	14.40 [13.11-15.77]	13.47 [12.39-14.62]	15.46 [14.62-16.32]	16.77 [15.88-17.69]
Heroin	negative	13.97 [12.86-15.14]	18.72 [17.28-20.21]	25.22 [23.79-26.70]	24.20 [22.58-25.89]	19.27 [17.48-21.16]	21.96 [20.28-23.72]	23.96 [22.25-25.74]
Heroin	positive	1.93 [1.76-2.11]	2.85 [2.61-3.10]	2.99 [2.75-3.25]	2.69 [2.42-2.99]	2.23 [1.94-2.55]	2.83 [2.55-3.13]	3.36 [3.04-3.70]
Heroin	total	3.47 [3.26-3.69]	5.01 [4.71-5.32]	6.53 [6.21-6.87]	6.43 [6.04-6.83]	4.99 [4.59-5.40]	5.73 [5.36-6.11]	6.85 [6.43-7.28]
Hydrocodone	negative	17.85 [16.70-19.04]	14.65 [13.45-15.92]	11.65 [10.66-12.70]	12.49 [11.23-13.84]	12.18 [10.67-13.83]	9.66 [8.46-10.97]	6.13 [5.19-7.19]
Hydrocodone	positive	3.04 [2.85-3.24]	3.18 [2.94-3.44]	2.63 [2.41-2.85]	3.02 [2.73-3.34]	2.72 [2.39-3.07]	1.96 [1.72-2.22]	1.64 [1.42-1.89]
Hydrocodone	total	4.89 [4.66-5.12]	4.78 [4.51-5.06]	4.06 [3.82-4.31]	4.63 [4.30-4.98]	4.18 [3.81-4.57]	3.11 [2.84-3.41]	2.39 [2.15-2.66]
Marijuana	negative	27.40 [24.12-30.88]	28.01 [26.30-29.78]	30.98 [29.37-32.62]	33.61 [31.66-35.61]	32.15 [29.91-34.46]	36.58 [34.47-38.74]	37.22 [35.13-39.34]
Marijuana	positive	23.29 [22.01-24.61]	22.37 [21.72-23.02]	23.74 [23.09-24.40]	25.99 [25.15-26.84]	26.59 [25.66-27.53]	29.48 [28.63-30.34]	31.12 [30.22-32.02]
Marijuana	total	23.89 [22.69-25.12]	23.17 [22.56-23.78]	24.91 [24.30-25.52]	27.35 [26.57-28.13]	27.48 [26.62-28.36]	30.57 [29.78-31.37]	32.13 [31.31-32.96]
Methadone	negative	7.71 [6.91-8.57]	6.49 [5.61-7.46]	5.59 [4.86-6.39]	6.41 [5.49-7.43]	5.50 [4.47-6.68]	5.25 [4.34-6.29]	3.85 [3.08-4.74]
Methadone	positive	1.52 [1.38-1.67]	1.66 [1.48-1.85]	1.36 [1.20-1.53]	1.31 [1.12-1.53]	1.26 [1.04-1.51]	1.07 [0.90-1.27]	1.13 [0.94-1.34]
Methadone	total	2.30 [2.14-2.47]	2.31 [2.11-2.53]	2.03 [1.85-2.22]	2.20 [1.97-2.45]	1.94 [1.69-2.22]	1.69 [1.49-1.92]	1.58 [1.38-1.81]
Methamphetamine	negative	3.03 [2.53-3.60]	3.60 [2.96-4.32]	7.21 [6.38-8.10]	11.71 [10.31-13.22]	15.78 [13.97-17.71]	26.98 [25.08-28.94]	31.62 [29.74-33.56]
Methamphetamine	positive	1.31 [1.18-1.45]	2.30 [2.09-2.52]	3.56 [3.30-3.83]	6.78 [6.30-7.29]	9.79 [9.16-10.44]	12.98 [12.38-13.60]	16.96 [16.28-17.67]
Methamphetamine	total	1.53 [1.40-1.66]	2.47 [2.27-2.69]	4.14 [3.88-4.41]	7.57 [7.10-8.05]	10.71 [10.10-11.34]	15.07 [14.48-15.67]	19.45 [18.79-20.12]
Oxycodone	negative	20.20 [18.99-21.46]	20.31 [18.91-21.76]	19.05 [17.82-20.33]	18.54 [17.07-20.08]	16.72 [15.02-18.53]	11.67 [10.37-13.07]	11.91 [10.62-13.29]
Oxycodone	positive	4.60 [4.36-4.85]	4.79 [4.50-5.10]	4.81 [4.52-5.12]	5.27 [4.89-5.67]	4.27 [3.87-4.70]	2.97 [2.69-3.28]	2.74 [2.45-3.05]
Oxycodone	total	6.54 [6.28-6.81]	6.92 [6.59-7.25]	7.08 [6.76-7.41]	7.54 [7.13-7.97]	6.24 [5.80-6.70]	4.27 [3.95-4.60]	4.28 [3.95-4.63]
Tramadol	negative	6.88 [4.62-9.79]	5.76 [4.74-6.93]	5.31 [4.46-6.27]	5.36 [4.45-6.39]	8.52 [7.15-10.04]	6.78 [5.70-7.99]	5.41 [4.47-6.48]
Tramadol	positive	2.44 [1.91-3.08]	2.16 [1.89-2.45]	2.00 [1.77-2.26]	1.99 [1.73-2.28]	1.97 [1.68-2.30]	1.63 [1.40-1.89]	1.16 [0.96-1.39]
Tramadol	total	3.00 [2.44-3.64]	2.68 [2.41-2.98]	2.54 [2.30-2.81]	2.59 [2.32-2.88]	3.01 [2.67-3.37]	2.41 [2.15-2.69]	1.89 [1.65-2.15]

Note: Non-prescribed and/or illicit positivity rates (%) for alcohol, benzodiazepines, cocaine, fentanyl, gabapentin, heroin, hydrocodone, marijuana, methadone, methamphetamine, oxycodone, and tramadol stratified by detection of prescribed buprenorphine. Percent positivity rates and 95% CI values are calculated by year. Total positivity was calculated without buprenorphine stratification.

© 2021 Saloner B et al. *JAMA Network Open.*

eTable 3. Comparison of Characteristics for UDT Specimens From the First and Second Specimen Sample Populations Collected Between January 1, 2013, and December 31, 2019

	Specimens Tested, No. (%)	
**Characteristic**	***1st Specimen***	***2nd Specimen***
Unique Patient Specimens	*150,000 (100.00%)*	*137,722 (100.00%)*
Sex		
Female	*67,893 (45.26%)*	*62,768 (45.58%)*
Male	*82,107 (54.74%)*	*74,954 (54.42%)*
Age, y		
18-24	*15,151 (10.10%)*	*14,747 (10.71%)*
25-34	*62,149 (41.43%)*	*57,922 (42.06%)*
35-44	*40,681 (27.12%)*	*37,088 (26.93%)*
45-54	*20,186 (13.46%)*	*17,756 (12.89%)*
55+	*11,593 (7.73%)*	*9,986 (7.25%)*
US Census Division		
East North Central	*40,033 (26.69%)*	*39,554 (28.72%)*
East South Central	*12,483 (8.32%)*	*11,164 (8.11%)*
Mid Atlantic	*25,494 (17.00%)*	*21,029 (15.27%)*
Mountain	*9,527 (6.35%)*	*8,274 (6.01%)*
New England	*9,666 (6.44%)*	*9,308 (6.76%)*
Pacific	*13,384 (8.92%)*	*11,864 (8.61%)*
South Atlantic	*30,398 (20.27%)*	*28,766 (20.89%)*
West North Central	*3,115 (2.08%)*	*2,964 (2.15%)*
West South Central	*5,900 (3.93%)*	*4,799 (3.48%)*
Health Care Practice Specialty		
Substance Use Treatment	*82,296 (54.86%)*	*80,496 (58.45%)*
Behavioral Health	*22,575 (15.05%)*	*19,042 (13.83%)*
Primary Care Physician	*45,129 (30.09%)*	*38,184 (27.73%)*
Payor Group		
Private insurance	*23,725 (15.82%)*	*23,430 (17.01%)*
Medicaid	*44,220 (29.48%)*	*45,173 (32.80%)*
Medicare	*7,279 (4.85%)*	*6,974 (5.06%)*
Uninsured	*12,395 (8.26%)*	*10,273 (7.46%)*
Unknown	*62,381 (41.59%)* [a]	*51,872 (37.66%)*
UDT Positives, No. (% positivity) [a]		
Buprenorphine	*128,240 (85.49%)*	*127,469 (92.56%)*
Benzodiazepines	*22,484 (18.87%)*	*18,148 (16.34%)*
Cocaine	*12,037 (8.87%)*	*10,072 (8.08%)*
Alcohol	*12,215 (14.67%)*	*10,873 (13.04%)*
Fentanyl	*5,217 (4.94%)*	*4,194 (4.18%)*
Gabapentin[b]	*3,773 (14.66%)*	*3,627 (13.76%)*
Heroin	*6,785 (5.38%)*	*4,438 (3.80%)*
Hydrocodone	*5,699 (4.19%)*	*2,608 (2.06%)*
Methadone	*2,667 (2.07%)*	*1,870 (1.57%)*
Methamphetamine	*8,740 (6.85%)*	*6,644 (5.65%)*
Marijuana	*24,564 (26.92%)*	*23,848 (26.29%)*
Oxycodone	*8,586 (6.30%)*	*4,770 (3.77%)*
Tramadol	*1,953 (2.53%)*	*1,341 (1.74%)*

Abbreviations: Urine Drug Testing (UDT)

© 2021 Saloner B et al. *JAMA Network Open.*

Notes:

150,000 randomly selected patient specimens collected between 2013 and 2019 and tested for prescribed buprenorphine were evaluated.

[a] All positive numbers and positivity rates are based on the non-prescribed analyte population except buprenorphine

[b] No gabapentin UDT results occurred prior to June 2014.

© 2021 Saloner B et al. *JAMA Network Open.*

eTable 4. Comparison of Logistic Regression Results for UDT Specimens From the First and Second Specimen Sample Populations

	**1st Specimen**			**2nd Specimen**		
	**Negative Probability**	**Positive Probability**	**Adjusted OR**	**Negative Probability**	**Positive Probability**	**Adjusted OR**
Benzodiazepines	0.247[0.236-0.258]	0.181[0.175-0.187]	1.48 [1.41-1.56]	0.184[0.171-0.198]	0.167[0.162-0.173]	1.12 [1.04-1.21]
Cocaine	0.121[0.113-0.129]	0.049[0.046-0.053]	2.64 [2.50-2.78]	0.103[0.094-0.113]	0.044[0.041-0.047]	2.51 [2.33-2.70]
Alcohol	0.175[0.165-0.187]	0.147[0.141-0.153]	1.24 [1.16-1.32]	0.146[0.133-0.161]	0.133[0.127-0.139]	1.12 [1.02-1.23]
Fentanyl	0.119[0.109-0.129]	0.020[0.018-0.022]	6.71 [6.29-7.16]	0.086[0.072-0.104]	0.018[0.015-0.021]	5.27 [4.85-5.73]
Gabapentin[a]	0.128[0.115-0.143]	0.123[0.115-0.133]	1.05 [0.94-1.15]	0.095[0.080-0.112]	0.108[0.100-0.117]	0.86 [0.74-1.00]
Heroin	0.143[0.133-0.154]	0.017[0.015-0.018]	9.93 [9.31-10.59]	0.122[0.109-0.135]	0.014[0.013-0.016]	9.60 [8.86-10.41]
Hydrocodone	0.114[0.105-0.123]	0.024[0.023-0.026]	5.11 [4.73-5.53]	0.064[0.055-0.073]	0.015[0.013-0.017]	4.47 [3.92-5.09]
Methadone	0.052[0.046-0.058]	0.011[0.010-0.013]	4.75 [4.26-5.29]	0.034[0.028-0.041]	0.010[0.008-0.011]	3.53 [3.03-4.09]
Methamphetamine	0.137[0.128-0.146]	0.069[0.066-0.073]	2.14 [2.01-2.27]	0.120[0.109-0.131]	0.059[0.056-0.063]	2.17 [1.99-2.36]
Oxycodone	0.142[0.133-0.152]	0.036[0.033-0.038]	4.50 [4.23-4.79]	0.087[0.078-0.098]	0.026[0.024-0.028]	3.61 [3.27-3.99]
Marijuana	0.309[0.298-0.321]	0.253[0.246-0.259]	1.33 [1.27-1.39]	0.263[0.248-0.278]	0.249[0.242-0.255]	1.08 [1.01-1.15]
Tramadol	0.055[0.049-0.062]	0.016[0.014-0.017]	3.69 [3.32-4.10]	0.033[0.027-0.040]	0.013[0.012-0.015]	2.57 [2.16-3.03]

Notes: Adjusted marginal probabilities and adjusted Odds Ratios for the 1st and 2nd specimen sample populations are shown

[a] Gabapentin aOR was not found to be significant in either the 1st or 2nd sample populations. All other aOR values were significant for both sample populations at the p<=0.05 level.

© 2021 Saloner B et al. *JAMA Network Open.*

eFigure. Positivity Rates for All Substances Stratified by Year and Patient Positivity for Buprenorphine

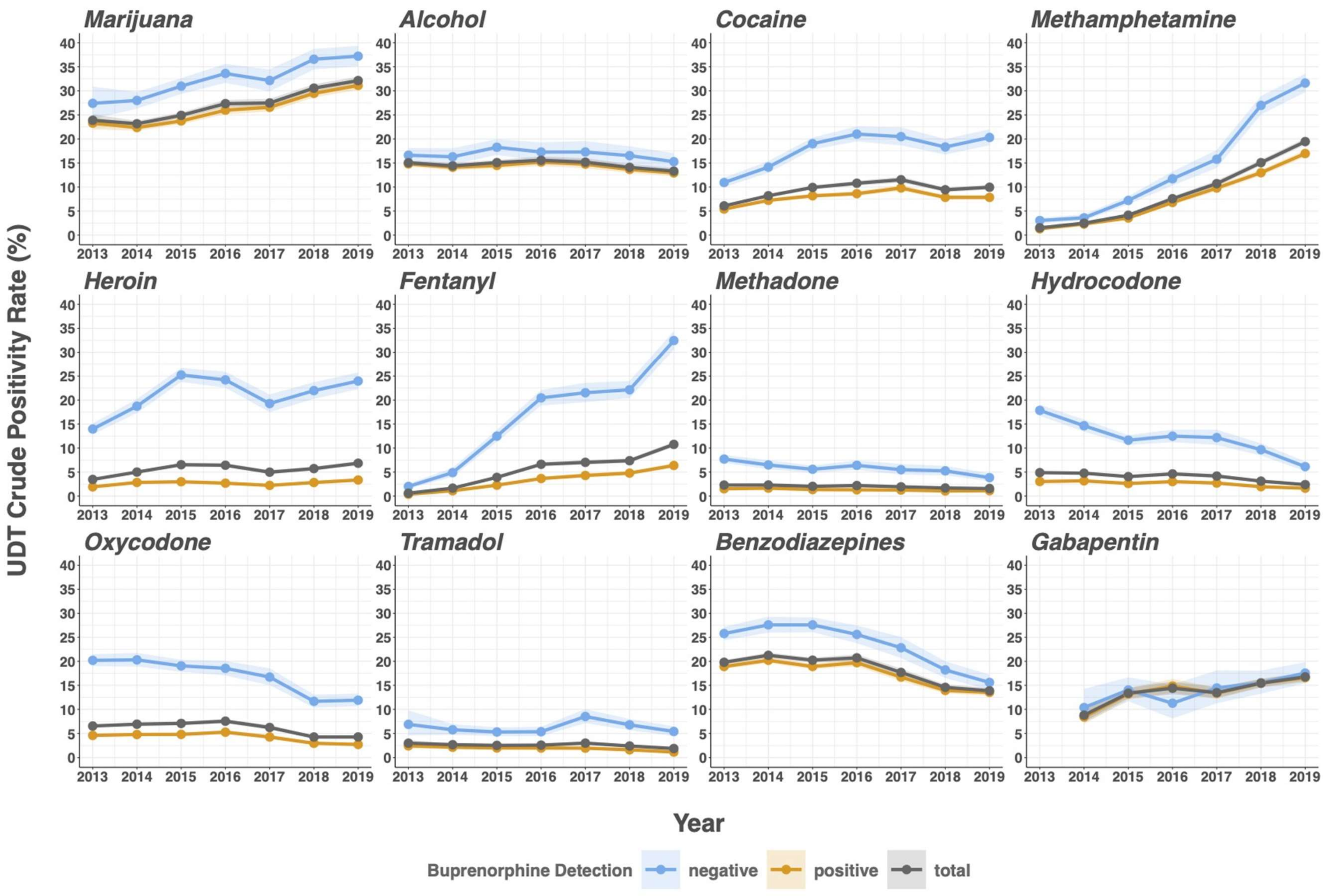

© 2021 Saloner B et al. *JAMA Network Open.*
